# Supplementary material for: High levels of SARS-CoV-2–specific T cells with restricted functionality in severe courses of COVID-19
Source: JCI Insight. 2020 Oct 15;5(20):e142167. doi: 10.1172/jci.insight.142167 (PMC7605520; doi:10.1172/jci.insight.142167)
Supplement: Supplemental data [file jciinsight-5-142167-s039.pdf]

Supplementary Information

High levels of SARS-CoV-2 specific T-cells with restricted functionality in severe course of COVID-19

David Schub[1], Verena Klemis[1], Sophie Schneitler[2], Janine Mihm[3], Philipp M. Lepper[4], Heinrike Wilkens[4], Robert Bals[4], Hermann Eichler[5], Barbara C. Gärtner[2], Sören L. Becker[2], Urban Sester[3], Martina Sester[1,*], and Tina Schmidt[1]

[1]Department of Transplant and Infection Immunology, [2]Institute of Medical Microbiology and Hygiene, [3]Department of Internal Medicine IV, [4]Department of Internal Medicine V, [5]Institute of Clinical Hemostaseology and Transfusion Medicine, Saarland University, 66421 Homburg, Germany

*Correspondence: Martina Sester, PhD, Saarland University, Department of Transplant and Infection Immunology, Institutes of Infection Medicine, building 47, Kirrberger Straße, D-66421 Homburg, Germany; email: martina.sester@uks.eu

Supplementary figures

Figure S1

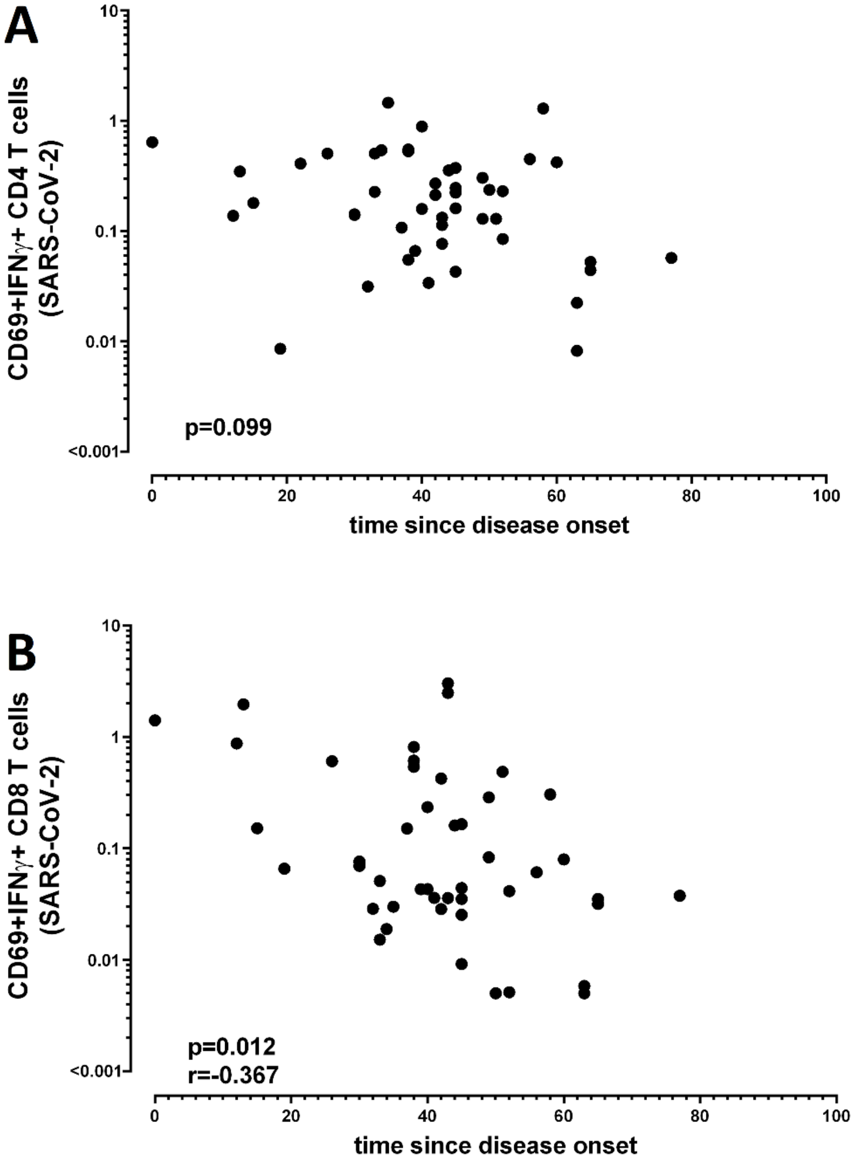

Figure S1: Levels of SARS-CoV-2 specific CD8 T cells inversely correlate with time since onset of disease. Total percentages of SARS-CoV-2 specific **(A)** CD4 and **(B)** CD8 T cells, determined by the sum of percentages towards the individual peptide pools for each patient, were correlated with time since onset of disease. Correlation was performed according to Spearman, $p<0.05$ was considered significant. IFN, interferon.

Figure S2

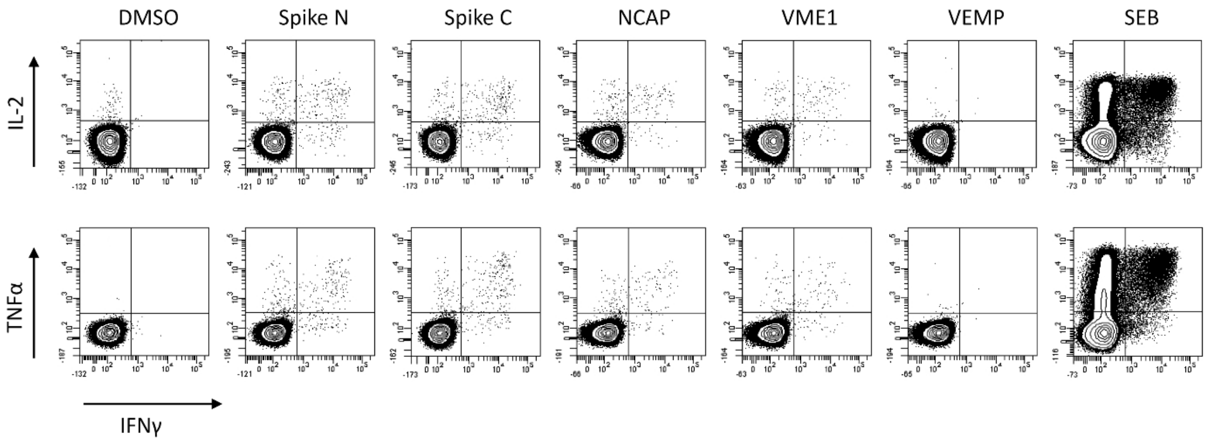

Figure S2: Cytokine expression of CD4 T cells after antigen-specific stimulation. Representative contour plots of CD4 T cells showing expression of the cytokines IL-2, TNFα and IFNγ after antigen-specific stimulation of a whole blood sample of a patient with severe COVID-19. IFN, interferon; IL, interleukin; TNF, tumor necrosis factor.

Figure S3

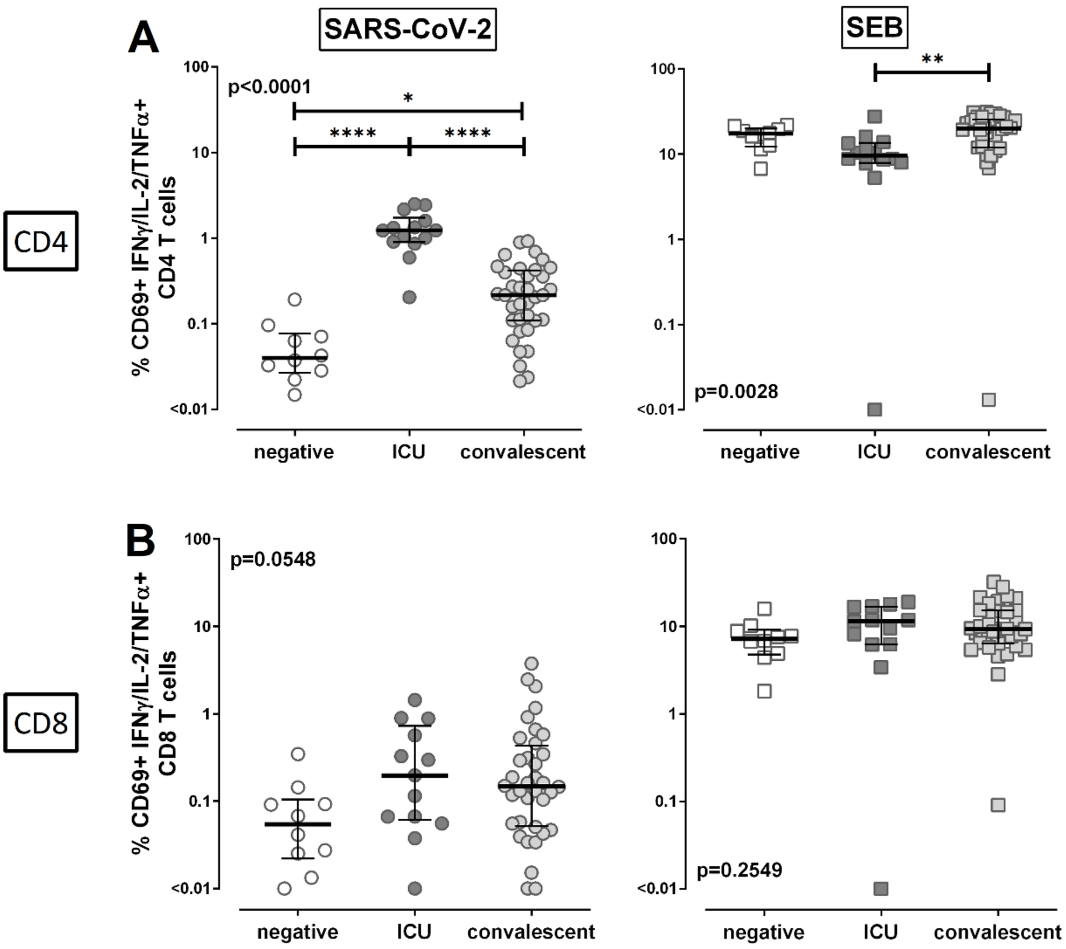

Figure S3: Patients with severe COVID-19 show increased levels of cytokine-positive SARS-CoV-2 specific CD4 T cells. Total percentages of SARS-CoV-2 specific and SEB-reactive **(A)** CD4 T cells and **(B)** CD8 T cells producing any of the three cytokines IFNγ, TNFα and IL-2 alone or in combination as determined by Boolean gating (gating strategy in figure S6, and schematic display in figure S8). The percentage of SARS-CoV-2 specific T-cells was determined as the sum of reactive cells towards the individual peptide pools for each individual. Results were compared between SARS-CoV-2 negative individuals (negative, n=10), patients with severe COVID-19 (ICU, n=14) and convalescent patients (n=36). Bars represent medians with interquartile ranges. Differences between the groups were calculated using Kruskal-Wallis test

and Dunn's post test. *P<0.05, **P<0.01, ****P<0.0001. IFN, interferon; IL, interleukin; TNF, tumor necrosis factor.

Figure S4

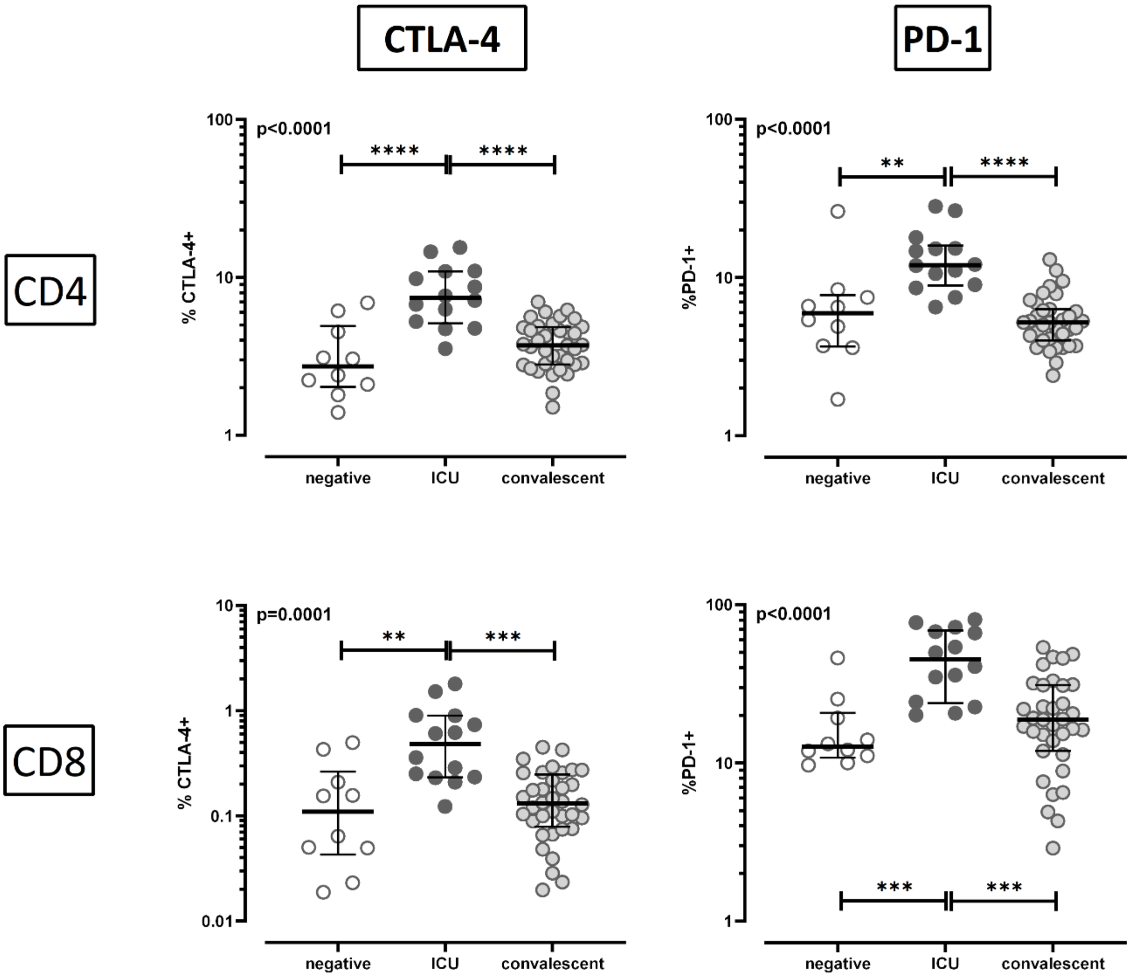

Figure S4: Altered percentage of CTLA-4 and PD-1 expressing CD4 and CD8 T cells in patients with severe COVID-19. Percentages of unstimulated total CD4 and CD8 T cells expressing cytotoxic T-lymphocyte antigen 4 (CTLA-4) or programmed cell death 1 (PD-1) were compared between SARS-CoV-2 negative individuals (negative, n=10), patients with severe COVID-19 (ICU, n=14) and convalescent patients (n=36). Bars represent medians with interquartile ranges. Differences between the groups were calculated using Kruskal-Wallis test and Dunn's post test. *P<0.05, **P<0.01, ***P<0.001, ****P<0.0001.

Figure S5

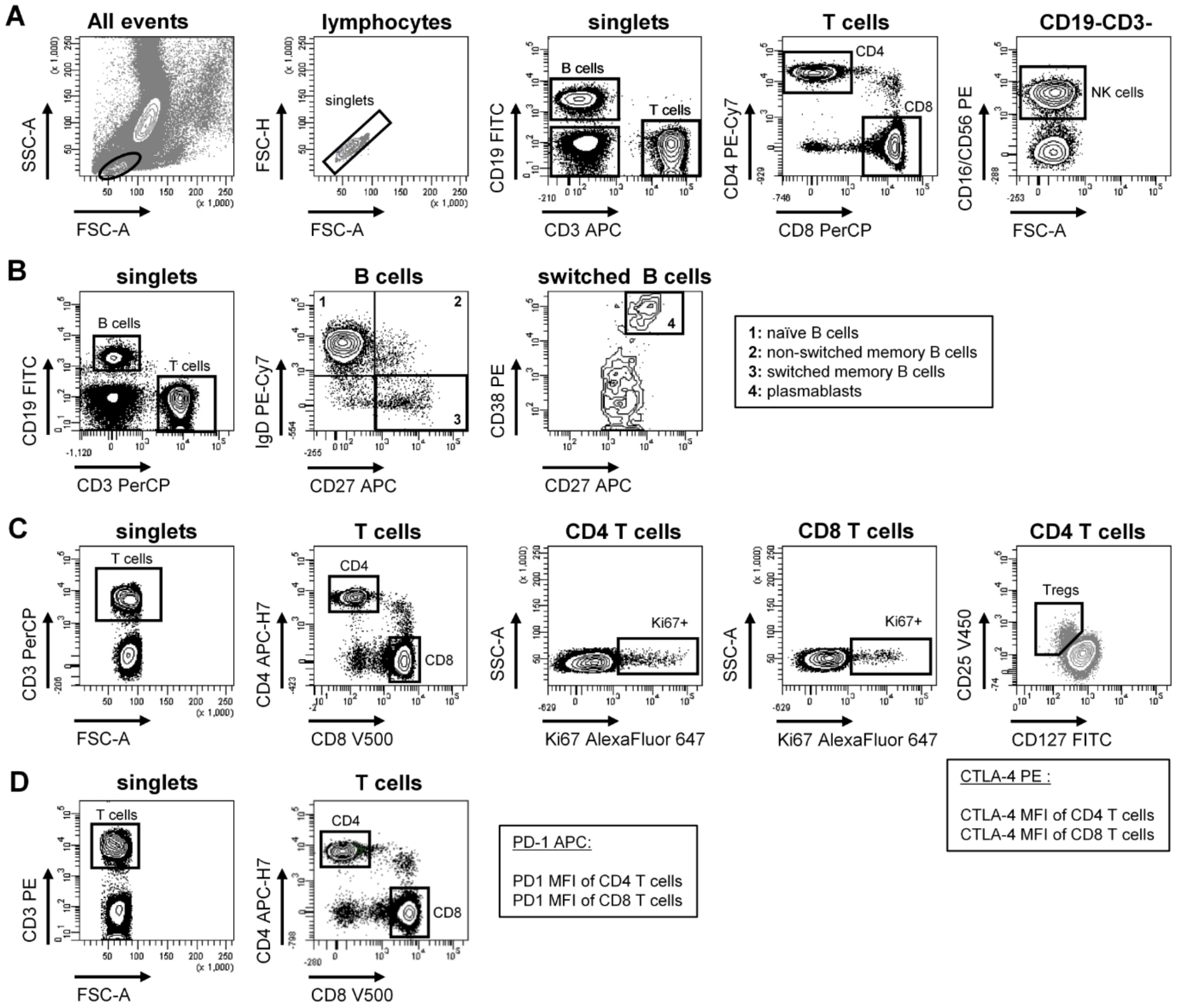

Figure S5: Gating strategies for quantitation of lymphocyte populations. (A) To quantify lymphocyte subpopulations, lymphocytes were gated within all events according to SSC-A and FSC-A. Exclusion of doublets was performed using hight and area signal of FSC. Among single lymphocytes, B cells and T cells were identified according to expression of CD19 and CD3. T cells were further subdivided into helper T cells (CD4) and cytotoxic T cells (CD8). NK cells were determined by CD16 and/or CD56 expressing cells among single lymphocytes neither expressing CD19 nor CD3. **(B)** Among single lymphocytes, B cells were identified as CD19+CD3- and T cells were gated according to expression of CD19 and CD3. According to IgD- and CD27-expression, B cells were subdivided in naïve (IgD+CD27-), non-switched memory (IgD+CD27+)

or switched memory B cells (IgD-CD27+). Plasmablasts were identified among switched memory B cells by additional staining of CD38. As plasmablasts may also be extended in size and granularity compared with non-plasmablast, lymphocyte gates (FSC and SSC) were enlarged for quantitation of these cells. **(C)** CD3+ T cells were identified among single lymphocytes and further subdivided in CD4 and CD8 T cells. Both subpopulations were analyzed towards expression of Ki67 (percentage of positive cells) and CTLA-4 (MFI of CD4 or CD8 T cells). Regulatory T cells (Tregs) were identified among CD4 T cells by low CD127 and high CD25 expression. **(D)** CD4 and CD8 T cells were identified among total T cells (CD3) and analyzed towards expression of PD-1 (PD1 MFI). CTLA-4, cytotoxic T lymphocyte antigen 4; FSC, forward scatter; PD-1, programmed cell death 1; SSC, side scatter.

Figure S6

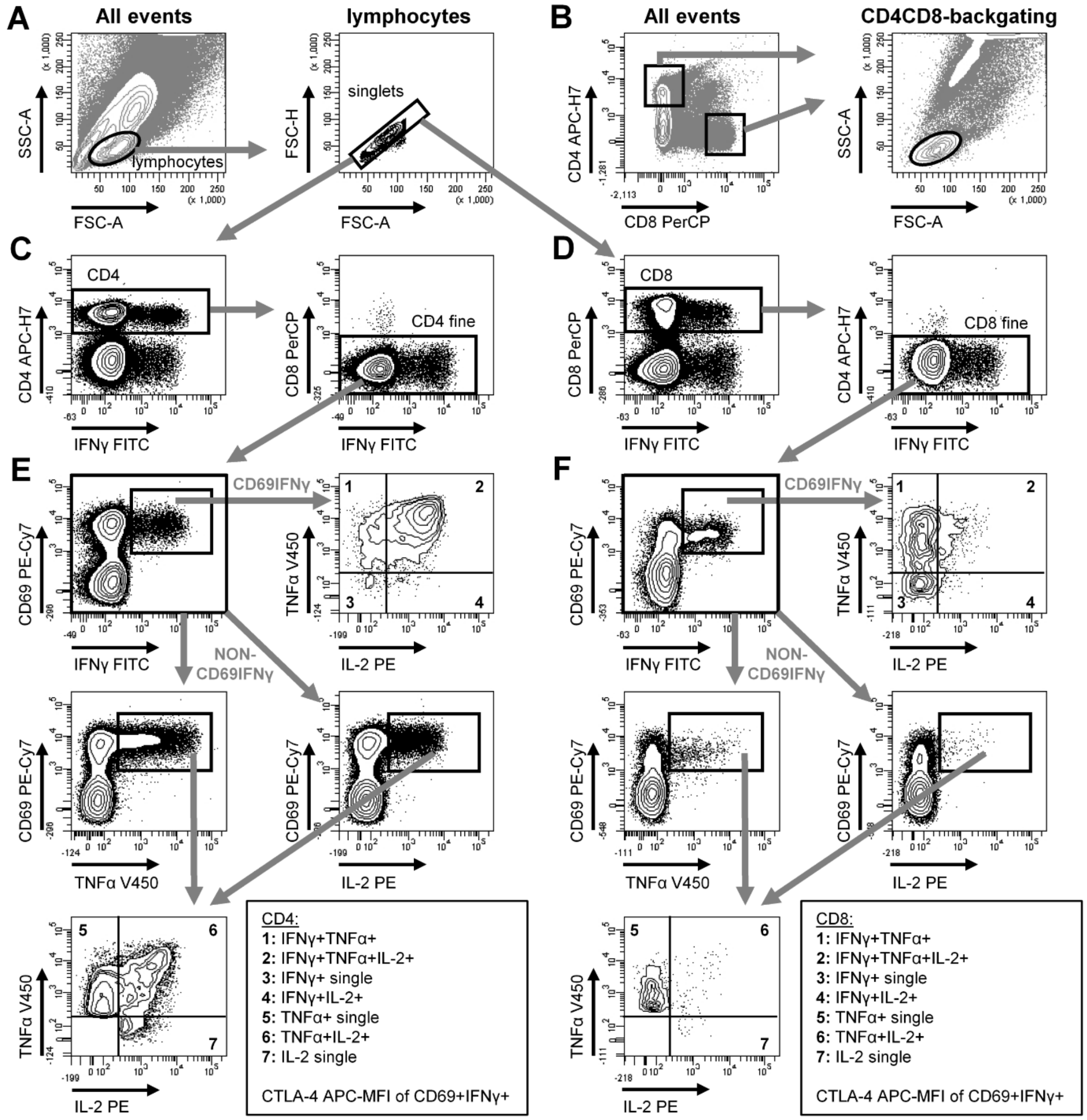

Figure S6: Gating strategy for analysis of cytokine and CTLA-4 expression of antigen-specific T cells. (A) Lymphocytes were gated among total events according to size (FSC) and granularity (SSC) followed by exclusion of doublets using hight and area signal of FSC. **(B)** To ensure accurate gating of lymphocytes, CD4/CD8 backgating was used which allows for an highlighted visualization of this population. **(C)** Among single lymphocytes, CD4 T cells (fine) were identified by exclusion of CD4-negative and CD8-positive cells. **(D)** Similarly, CD8 T cells (fine)

were identified among single lymphocytes by exclusion of CD8-negative and CD4-positive cells. To determine the percentage of antigen-specific cells, CD69+IFNγ+ T cells were identified among CD4 **(E)** and CD8 T cells **(F)**. CD69+IFNγ+ CD4 or CD8 T cells were subdivided according to expression of TNFα and IL-2. Among CD4 or CD8 T cells not coexpressing CD69 and IFNγ, „AND/OR-gating“ of CD69+TNFα+ and CD69+IL2+ CD4 or CD8 T cells allowed for identification of cells with single or combined expression of TNFα and IL-2. In addition expression of CTLA-4 (MFI) of CD69+IFNγ+ CD4 and CD8 T cells was analyzed. IFN, interferon; IL, interleukin; TNF, tumor necrosis factor.

Figure S7

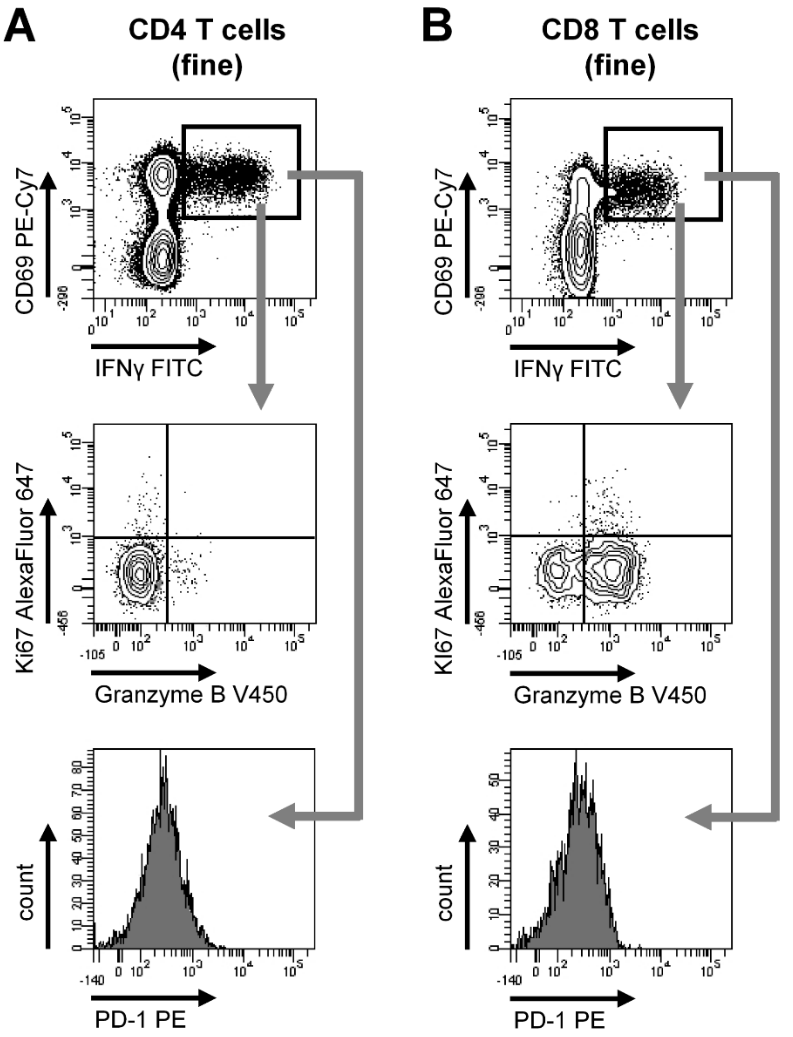

Figure S7: Gating strategy for analysis of Ki67-, Granzyme B- and PD-1-expression of antigen-specific T cells. Among CD4 **(A)** and CD8 T cells **(B)** (fine gating s. supplementary figure S2), antigen-specific cells were identified by co-expression of CD69 and IFNγ. These cells were analyzed towards their expression of Ki67 and/or Granzyme B (percentage of positive cells) as well as expression of PD-1 (MFI). IFN, interferon; PD-1, programmed cell death 1.

Figure S8

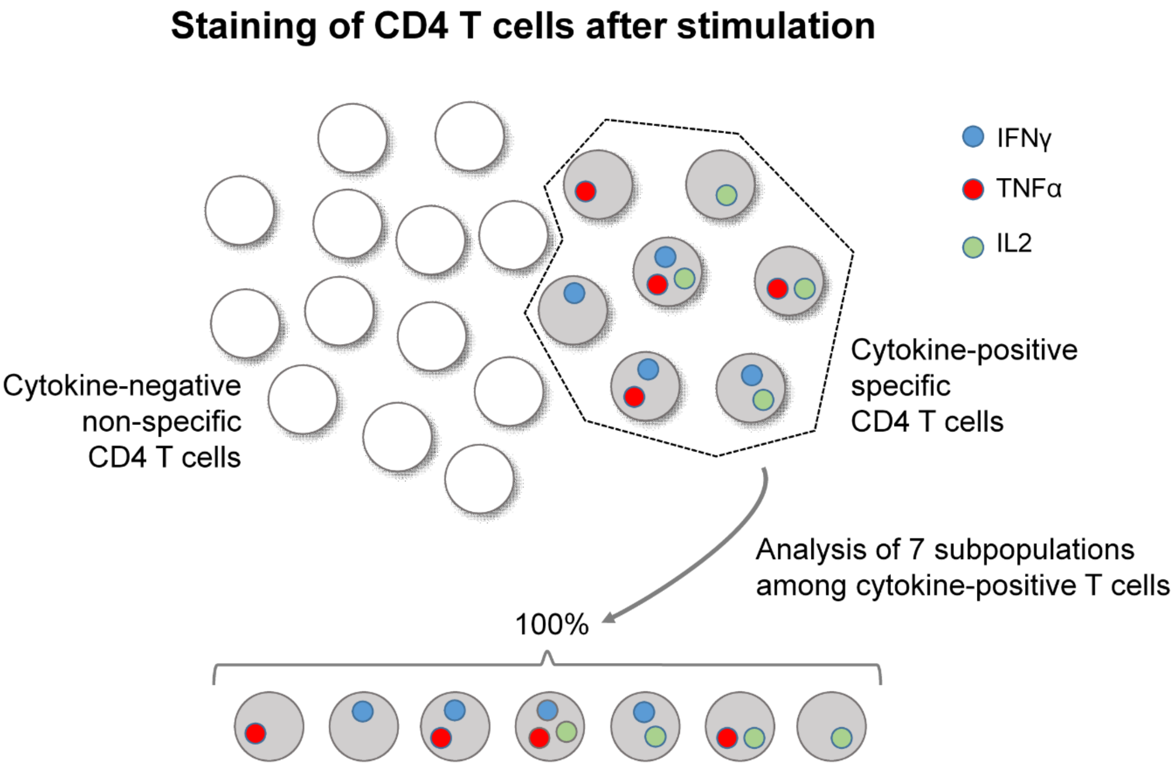

IFNγ		+	+	+	+		
TNFα	+		+	+		+	
IL-2				+	+	+	+

Figure S8: Determination of cytokine expression profiles of cytokine-positive specific CD4 T cells after stimulation. Cytokine-positive specific cells were determined and quantified among total CD4 T cells after antigen-specific stimulation (relevant for figure 2 (IFNγ-expressing cells in blue) and supplementary figure S3 (all cytokine-positive cell populations)). These cytokine-positive specific CD4 T cells were set as 100% and distribution of the 7 different subpopulations among these cytokine-positive specific CD4 T cells were analyzed and compared between patient groups (relevant for figure 3A). The schematic illustration does not indicate CD69 positivity as an additional marker for activated cells. IFN, interferon; IL, interleukin; TNF, tumor necrosis factor.

Supplementary Tables

Table S1: Antibodies for co-stimulation and flow cytometric analysis

Antigen	conjugate	clone	isotype	reactivity
CD3	APC, PerCP	SK7	IgG1 k	mouse anti-human
CD4	PE-Cy7, APC-H7	SK3	IgG1 k	mouse anti-human
CD8	V500, PerCP	RPA-T8, SK1	IgG1 k	mouse anti-human
CD16	PE	3G8	IgG1 k	mouse anti-human
CD19	FITC	HIB19	IgG1 k	mouse anti-human
CD25	V450	M-A251	IgG1 k	Mouse anti-human
CD27	APC	L128	IgG1 k	mouse anti-human
CD28*	none	L293	IgG1 k	mouse anti-human
CD38	PE	HB7	IgG1 k	mouse anti-human
CD49d*	none	9F10	IgG1 k	mouse anti-human
CD56	PE	B159	IgG1 k	mouse anti-human
CD69	PE-Cy7	L78	IgG1 k	mouse anti-human
CD127	FITC	eBioRDR5	IgG1 k	mouse anti-human
CD152 (CTLA-4)	PE, APC	BNI3	IgG2a k	mouse anti-human
CD279 (PD-1)	APC, PE	MIH4	IgG1 k	mouse anti-human
Granzyme B	V450	GB11	IgG1 k	mouse anti-human
IFNγ	FITC	4S.B3	IgG1 k	mouse anti-human
IgD	PE-Cy7	IA6-2	IgG2a k	mouse anti-human
IL-2	PE	MQ1-17H12	IgG2a k	rat anti-human
Ki67	AlexaFluor 647	B56	IgG1 k	mouse anti-human
TNFα	V450	MAb11	IgG1 k	mouse anti-human

All antibodies except for CD127 (eBioscience/Thermofisher) were purchased from BD.
* co-stimulatory antibodies

Table S2: SARS-CoV-2 peptide pools for antigen-specific stimulation

SARS-CoV-2 antigens	Protein ID	No. of peptides	Peptide sequence	Protein sequence covered*
Spike glycoprotein	P0DTC2	158 (N)<br>157 (C)	15mers with 11 aa overlap<br>+ last peptide (=17mer)	(N) 1-643 aa<br>(C) 633-1273 aa
NCAP	P0DTC9	102	15mers with 11 aa overlap	419 aa
VEMP	P0DTC4	16	15mers with 11 aa overlap	75 aa
VME1	P0DTC5	53	15mers with 11 aa overlap	222 aa

*each pool of peptides covers the total aminoacid (aa) sequence of the corresponding protein.
