# Supplementary material for: High levels of SARS-CoV-2–specific T cells with restricted functionality in severe courses of COVID-19
Source: JCI Insight. 2020 Oct 15;5(20):e142167. doi: 10.1172/jci.insight.142167 (PMC7605520; doi:10.1172/jci.insight.142167)
Supplement: Supplemental [file jciinsight-5-142167-s040.pdf]

General editorial comments

1. ***Ensure that your manuscript title conforms to JCI Insight's strict word limit. JCI Insight strongly recommends adhering to a 10-word title, and titles may not exceed 15 words.***

The title was now shortened to include 15 words as suggested.

2. ***The published author list will appear as presented in your submitted Word document. Please confirm that all author names are correct and complete.***

Confirmed.

3. ***Provide complete correspondence information -- mailing address (including street address or mail stop code), phone number, and email address -- for each corresponding author on the manuscript title page.***

This information has been added.

4. ***If appropriate, add a footnote on the manuscript title page specifying the "Role of funding source."***

The funding sources had no role in the conduct, analysis or interpretation of data.

5. ***When describing demographics of study participants, provide inclusive details on sex, race, and other factors; e.g., do not present values only for male, or only for White, participants; or categorize groups of people as "other." If this information is not available or incomplete, briefly explain why. Specify whether the participants or investigators made the classifications.***

We have provided inclusive data and classifications were done by the investigators based on anamnestic information by the study participants.

6. ***Per JCI Insight style for Clinical medicine submissions, revise the Abstract to follow a structured format, with the following headings: Background, Methods, Results, Conclusion, Trial registration, Funding. See http://insight.jci.org/kiosks/authors#Structured_Abstract for details.***

The Abstract was re-structured accordingly.

7. ***Ensure that protein and gene symbols are formatted correctly and are consistent with NCBI Gene nomenclature. See Gene Nomenclature and Style for details: http://insight.jci.org/kiosk/publish/genestyle.***

Not applicable.

8. ***Per JCI Insight style, provision of materials is considered part of the study methodology. Therefore, edit to cite all gifts of materials in Methods where appropriate, providing each individual's affiliation and location (including city).***

Not applicable.

9. ***Move all details of human and animal study approval and informed consent to a separate "Study approval" section at the end of Methods (following the Statistics overview).***

Done.

10. ***For all antibodies used, ensure that the source and catalog/clone number (in the case of commercial antibodies) or a description/reference to a description of the generation of custom antibodies is provided.***

Done. All antibodies are commercially available and respective information is given in the supplementary document (table S1).

11. ***Ensure that the source of all cell lines/animals used is indicated.***

Not applicable.

12. ***Because JCI Insight policy prohibits withholding methodology, your mention of methods being available upon request is insufficient. Edit to provide the relevant methodology details in full, either in Methods or Supplemental Methods; or as a table or supplemental table.***

All completely mentioned.

13. ***Ensure that all references are present, that there are no duplicates, and that the reference list is formatted according to JCI Insight style (http://insight.jci.org/kiosk/publish/examples). JCI Insight strongly encourages authors to use EndNote in Microsoft Word to ensure that the format is correct. Furthermore, limit the use of URLs within the main text to citation of online databases and similar websites; if the URL refers to a publication, convert it to a numbered reference with complete bibliographic details.***

Done.

14. ***Please ensure that all figures are prepared according to JCI Insight style (https://insight.jci.org/kiosk/publish/figures); in particular, figures should be presented on portrait-oriented pages, labels should be in 8 pt Helvetica or Arial font, and no bold type should be used.***

Done.

14. ***Include a CONSORT diagram as its own figure in the main text, ideally as Figure 1 (cited first).***

Not applicable, as no clinical study.

15. ***Ensure that the figure legends include, as applicable: (a) the statistical test used for the figure; (b) the exact number of samples (n) for each figure panel representing multiple experiments; and (c) in the case of representative experiments, the number of times the experiment was conducted.***

Done.

16. ***Ensure that quantitative data graphs are presented as dot plots, with the average and the appropriate error bars indicated; or as box-and-whisker plots, with values defined in the legend (the bounds of the boxes, the lines within the boxes, the whiskers, and any outlying values).***

Done.
